# Neuroprotection by remote ischemic conditioning in the setting of acute ischemic stroke: a preclinical two-centre study

**DOI:** 10.1038/s41598-020-74046-4

**Published:** 2020-10-09

**Authors:** Maryna V. Basalay, Marlene Wiart, Fabien Chauveau, Chloe Dumot, Christelle Leon, Camille Amaz, Radu Bolbos, Diana Cash, Eugene Kim, Laura Mechtouff, Tae-Hee Cho, Norbert Nighoghossian, Sean M. Davidson, Michel Ovize, Derek M. Yellon

**Affiliations:** 1 The Hatter Cardiovascular Institute, University College London, 67 Chenies Mews, London, WC1E 6HX UK; 2 Univ Lyon, CarMeN Laboratory, INSERM 1060, INRA U1397, INSA Lyon, Université Claude Bernard Lyon 1, Oullins, France; 3 Univ Lyon, Lyon Neuroscience Research Center, CNRS UMR5292, INSERM U1028, Université Claude Bernard Lyon 1, Lyon, France; 4 Clinical Investigation Center, CIC 1407, HCL, Louis Pradel Hospital, Lyon, France; 5 CERMEP-Imagerie du Vivant, Lyon, France; 6 Department of Neuroimaging, Institute of Psychiatry, Psychology and Neuroscience, King’s College London, James Black Centre, 125 Coldharbour Lane, London, SE5 9NU UK; 7 Univ Lyon, CREATIS, CNRS UMR 5220, INSERM U1206, INSA-Lyon, Université Claude Bernard Lyon 1, Lyon, France; 8 Stroke Department, Hospices Civils de Lyon, Lyon, France

**Keywords:** Cell biology, Neuroscience, Physiology, Neurology

## Abstract

Reperfusion is the only existing strategy for patients with acute ischemic stroke, however it causes further brain damage itself. A feasible therapy targeting reperfusion injury is remote ischemic conditioning (RIC). This was a two-centre, randomized, blinded international study, using translational imaging endpoints, aimed to examine the neuroprotective effects of RIC in ischemic stroke model. 80 male rats underwent 90-min middle cerebral artery occlusion. RIC consisted of 4 × 5 min cycles of left hind limb ischemia. The primary endpoint was infarct size measured on T2-weighted MRI at 24 h, expressed as percentage of the area-at-risk. Secondary endpoints were: hemispheric space-modifying edema, infarct growth between per-occlusion and 24 h MRI, neurofunctional outcome measured by neuroscores. 47 rats were included in the analysis after applying pre-defined inclusion criteria. RIC significantly reduced infarct size (median, interquartile range: 19% [8%; 32%] vs control: 40% [17%; 59%], *p* = 0.028). This effect was still significant after adjustment for apparent diffusion coefficient lesion size in multivariate analysis. RIC also improved neuroscores (6 [3; 8] vs control: 9 [7; 11], *p* = 0.032). Other secondary endpoints were not statistically different between groups. We conclude that RIC in the setting of acute ischemic stroke in rats is safe, reduces infarct size and improves functional recovery.

## Introduction

Currently, ischemic stroke remains one of the most costly and devastating clinical syndromes in the world^1,2^. Recently, endovascular recanalization with mechanical thrombectomy (MT) has brought about a paradigm shift in the optimal management of patients with large vessel occlusion^3,4^. However, despite a successful recanalization achieved in more than 70% of cases, functional independence (modified Rankin score mRs 0–2 at 3 months after ischemic stroke) was obtained only in nearly 45% of patients treated with MT + /− intravenous tissue recombinant plasminogen activator^5^. This reveals the need to develop new adjunctive neuroprotective strategies alongside reperfusion therapy.

Although reperfusion is the prerequisite to salvage ischemic tissue, restoring cerebral circulation may paradoxically cause further damage to jeopardised tissue. Though mostly studied in the heart^6–8^, reperfusion injury has also been suggested in the brain^9–13^. As such, targeting reperfusion injury should be considered an effective means of developing additional adjunctive therapies in patients with acute ischemic stroke^14,15^. Among the therapies targeting reperfusion injury, a potentially feasible one is remote ischemic conditioning (RIC)—a method whereby the application of brief episodes of ischemia and reperfusion to an organ/tissue can protect a remote organ (the heart or the brain) from subsequent ischemic injury^16^. This strategy is currently being tested in 5 on-going clinical trials in patients with ischemic stroke (https://clinicaltrials.gov). However, to date, the two completed trials^17–19^ did not show a benefit in such patients. This may question the results of the numerous single-centre animal studies that demonstrated an infarct-limiting effect of RIC in acute stroke models (reviewed in^20,21^).

Extensive experimental research over many years has identified neuroprotective therapies that appeared promising in single-centre animal studies. However, none of these neuroprotective agents have been successful in clinical trials. The possible explanations for this uncertain record of translation have been extensively discussed, e.g.: at the Stroke Treatment Academic Industry Roundtable (STAIR)^22^. These and subsequent discussions produced guidelines with strong recommendations for the study of potential neuroprotective therapies^23^. In this regard, none of the published experimental studies on the effect of RIC in acute ischemic stroke models^18,20,21^ sufficiently fulfil the requirements of STAIR for effective translational research^22^. Recent meta-analysis of RIC in experimental stroke revealed that the median study quality was only 7 (range 4–9/10)^21^. Moreover, significant publication bias has been identified (*p* < 0.001)^21^, strongly justifying the need for a well-powered and unbiased study. In addition, one of the major requirements of STAIR guidelines is for collaboration, stating that the study should be performed in two or more laboratories^22,24^. International collaboration has been identified as a factor providing high levels of complementarity in pre-clinical stroke studies^25^.

Therefore, the main objective of this study was to test the neuroprotective effects of RIC in a rat model of acute ischemic stroke in a two-centre, international study, using translational imaging endpoints.

## Materials and methods

All experiments were performed in accordance with the European Commission Directive 2010/63/EU (European Convention for the Protection of Vertebrate Animals used for Experimental and Other Scientific Purposes), as well as the French decree 2013-118 and the UK Home Office (Scientific Procedures) Act (1986) with project approval from the respective Institutional Animal Care and Use Committees. All procedures regarding the study design, animal experiments, statistical analysis, and data reporting fulfilled the criteria of the ARRIVE (Animal Research: Reporting in Vivo Experiments) guidelines^26^.

### Animals used

Eighty Sprague–Dawley male rats (strain Crl:CD(SD)) weighing 250–300 g (Charles River Laboratories) were used.

### Animals and experimental groups

The animals were supplied in several consecutive batches. After arrival to a Biological Service Unit, they were allowed to acclimatize for at least 7 days before use, being housed in a controlled temperature (22 ± 2 °C) with a 12/12-h light/dark cycle and access to pelleted food and water ad libitum. The number of animals per cage, the use of environmental enrichment and food type were standard for each of the research groups, in accordance with local regulatory requirements. In London, the study protocol was ethically reviewed by the KCL Animal Welfare Review Body (AWERB). In Lyon, the protocol was approved by the local ethical review board (“Comité d’éthique pour l’Expérimentation Animale Neurosciences Lyon”, registration code: C2EA—42) and authorized by the French Ministry of Higher Education and Research (n°15529-2018061512184831v2).

### Study design

The overall sample size for the study was calculated using the R statistical software version 3.6.1. An alpha level of 0.05 and a power of 0.8 were required. Expecting 40% reduction of infarct volume in the treatment group and variability of 40% based on our previously published data^27^, the minimal total number of animals to be included was determined as 34, i.e. 17 in each experimental group using the two-tailed Wilcoxon–Mann–Whitney tests for two groups.

The study was randomized and blinded. Randomization was performed by block of 4 animals per day (i.e. 2 animals were randomly allocated to the 2 experimental groups, RIC or control, each day) using a randomization table generated by GraphPad online calculator QuickCalcs (https://www.graphpad.com/quickcalcs/randomize1.cfm).

Supplementary Fig. S1 shows the experimental design. In brief, rats were subjected to transient middle cerebral artery occlusion (MCAO) using the intraluminal filament model. MRI was first performed per-occlusion using the same MRI protocol as for acute stroke patients at admission (adapted at 7 T): MR angiography (MRA), T2-weighted imaging (T2WI), diffusion-weighted imaging (DWI) and perfusion-weighted imaging (PWI). The primary purpose of per-occlusion MRI was: (1) to exclude animals with no focal cerebral ischemia; and (2) to quantify baseline DWI and PWI lesion volumes. Reperfusion was obtained outside the magnet after 90 min of MCAO. RIC treatment (4 cycles of 5-min left hind-limb ischemia interleaved with 5-min reperfusion) was started 10 min before reperfusion. A follow-up MRI scan was obtained for each animal at the end of the 24 h recovery period, after being rated for neurofunctional outcome and before being euthanized. This second MRI aimed primarily at evaluating the infarcted area and the extent of space-modifying edema in vivo.

The predefined operational exclusion criteria were: the absence of a lesion on DWI and/or the absence of a perfusion defect on PWI, the presence of hemorrhage on T2WI, the death of an animal before treatment, the poor quality of MR images. The predefined analytical exclusion criteria was the failure to obtain a complete follow-up until the time of sacrifice (because the animal died between treatment and 24 h or because MRI data were not interpretable). Before unblinding the results, an additional analytical exclusion criterium was defined for rats having an edema-corrected infarcted volume exceeding the per-occlusion hypoperfused volume by more than 20%. Our hypothesis was that the hypoperfused region represents the tissue that will get infarcted if reperfusion is not timely obtained. According to this model, the edema-corrected final infarct cannot excessively exceed the per-occlusion hypoperfused area. The per-occlusion hypoperfused area is hereafter referred to as the ‘area at risk’ of infarction (AAR). Of note, this terminology is adopted in analogy to myocardial ischemia studies in which the AAR comprises the hypoperfused region that gets infarcted in case of permanent occlusion. It should not be mistaken with the DWI–PWI mismatch or ischemic penumbra, often referred to as ‘tissue at risk’ in the stroke community, with the assumption that the per-occlusion DWI lesion represents irreversible brain damage. In the present study, the AAR was used to normalize the final infarct size for each animal to account for inter-individual variability in ischemic areas generated by MCAO.

Data from all included animals in both centres were pooled for the final analysis. A subgroup analysis was further performed including animals with corticostriatal lesions only (defined as per-occlusion DWI lesion > 100 mm^3^). Safety data consisted in counting the number of animals that either died post-treatment or underwent hemorrhagic transformation in each group. The predetermined primary endpoint of the study was infarct size (IS) measured on T2WI at 24 h, corrected for edema, and expressed as percentage of the AAR for each animal individually. The perfusion defect, representing AAR, was detected visually, based on asymmetry of perfusion compared to the contralateral hemisphere. The secondary endpoints were: (1) hemispheric space-modifying effect of edema (%HSE) estimated on MRI; (2) infarct growth estimated on MRI, i.e. the difference between H24 T2WI lesion volume and per-occlusion DWI lesion volume; and (3) neurofunctional outcome. Lesion sizes were calculated in mm^3^ by taking into account the voxel size for each sequence. In addition, we analyzed lesion volumes on the photos of brain slices after triphenyl tetrazolium chloride (TTC) staining and compared the final infarct volumes as evaluated by TTC and MRI.

### Transient middle cerebral artery occlusion

The intraluminal filament model of focal ischemia was used^28,29^. Briefly, under 2% isoflurane anesthesia, a size-matched silicon-coated monofilament (Doccol Corporation, USA) was advanced through the right internal carotid artery towards the middle cerebral artery (MCA) junction until resistance was felt (~ 2 cm). The filament was withdrawn after 90 min of occlusion.

### Magnetic resonance imaging

The animals were placed in supine position in a dedicated plastic cradle and maintained during the entire MRI protocol under anaesthesia at 1.5–2.5% isoflurane delivered via a cone mask. A respiratory sensor and rectal temperature probe were used to continuously monitor the respiration rate and body temperature (Rapid Biomedical or SA Instruments). Body temperature was maintained at 36.5–37.0 °C by means of a feedback-regulated air heater (in London) or a heated water circuit integrated within the dedicated bed (in Lyon).

Supplementary Table S1 and Table S2 provide the characteristics of each system and the sequence parameters used in each centre.

### Remote ischemic conditioning

RIC was performed as 4 cycles of 5-min left hind limb ischemia^30^ interleaved with 5-min reperfusion, using an inflatable 12-mm cuff (IVM, USA), which was inflated to 200 mmHg and subsequently deflated. The efficiency of blood cessation with hind limb cuff inflation in rats had previously been confirmed^31^. Of note, because of its mechanical nature, the allocation of rat to treatment group could not be concealed from the investigator performing the RIC treatment (M.B. in both centres).

### Functional status evaluation

Behavioural neurological evaluation was performed 24 h after the onset of reperfusion using the 0–22 scale score, which incorporated three previously reported scales^32–35^. The principal points of functional status evaluation were: spontaneous activity, gait, postural signs, lateral resistance, limb placing, and parachute reflex (Table S3). Higher neuroscores reflected stronger neurological deficits. Neurofunctional evaluation was initially done by two researchers (one senior F.Ch. and one junior M.B.) for the first 10 animals, and the agreement was reached on each point. Subsequently, neurofunctional evaluation in both centres was performed by one researcher (M.B.) who was blinded on treatment allocation. With the purpose of blinding, the animals were delivered by another researcher to a separate room, where the evaluation was performed, with ID concealment and in random order.

### Triphenyl tetrazolium chloride staining

TTC staining was used to assess histological necrosis. Immediately after the second MRI, the animals were euthanized with overdosage of isoflurane. The brains were quickly extracted, sectioned at 1.5-mm intervals, stained with 1% TTC for 15 min and fixed in formalin. Sections were photographed, and the infarcted areas were measured blindly using ImageJ software (https://imagej.nih.gov/ij/).

### MRI analysis

All the MRI analyses were done in a blinded manner with respect to treatment group allocation, neurofunctional outcome and TTC staining. All MR images were independently analyzed by two researchers (one junior M.B. and one senior M.W.) who manually delineated the areas of interest detailed thereafter using ImageJ software for the two centres.

Maximum intensity projection analysis of angiography images was used to assess MCA occlusion. An angiographical score was defined for semiquantitative analysis^35^:

- 0 = undetectable flow along the whole MCA route (complete occlusion);
- 1 = decreased flow signal (partial occlusion);
- 2 = normal flow signal (no occlusion).

Quantitative apparent diffusion coefficient (ADC) maps were obtained from the monoexponential fitting of the signal versus b-value for each slice of the DWI sequence. This post-processing was done within MATLAB environment (https://uk.mathworks.com/products/matlab.html)^36^ as described previously^37^. PWI dynamic series were also post-processed off-line using a homemade MATLAB code described previously in order to obtain semi-quantitative perfusion maps (maximum peak concentration or MPC).

Supplementary Fig. S2 shows typical examples of the longitudinal follow-up of one excluded animal and two included ones: one in the control group and one in the RIC group. The data obtained from the analysis of the MR images were the volumes of: ADC lesion, AAR (measured as perfusion defect on MPC map), infarcted area (uncorrected lesion volume, LVu) and ipsilateral and contralateral total hemispheres on follow-up T2WI. Hemispheric space-modifying effect of edema was calculated as: %HSE = 100% * (ipsilateral hemisphere volume–contralateral hemisphere volume)/contralateral hemisphere volume^38,39^. Lesion volume was corrected for edema (corrected lesion volume, LVc) using Gerriets correction^38,39^. IS was expressed as % of the AAR to account for inter-individual variability in ischemic areas following MCAO. Supplementary Fig. S2 demonstrates, that one animal from the control group did not have MRA data for technical reasons. MRA showed complete or partial MCAO in 20 of the 22 animals with MRA in the control group (91%) and 22 of 24 animals in the RIC treatment group (92%) with no significant difference (*p* = 0.801).

### Statistical analysis

Statistical analysis was performed by an independent professional statistician (C.A.) using the R statistical software version 3.6.1^40^. The Shapiro–Wilk test was used to evaluate normality of the data. Bivariate comparisons were made using Student’s t test for continuous variables or Wilcoxon test when normality assumptions were not validated. X^2^ tests (or Fischer’s exact test when the expected cell frequency was < 5) were used for categorical variables (MRA score and neuroscores). The difference in ADC lesion sizes at baseline was taken into account in a multivariate linear regression. Differences between groups were considered statistically significant when *p* < 0.05. The agreement between the measurements obtained for the same variables by two researchers and by two methods (MRI and TTC staining) was analyzed using Intraclass Correlation Coefficient (ICC). Data are presented as median [25% percentile; 75% percentile].

## Results

**Figure 1:**
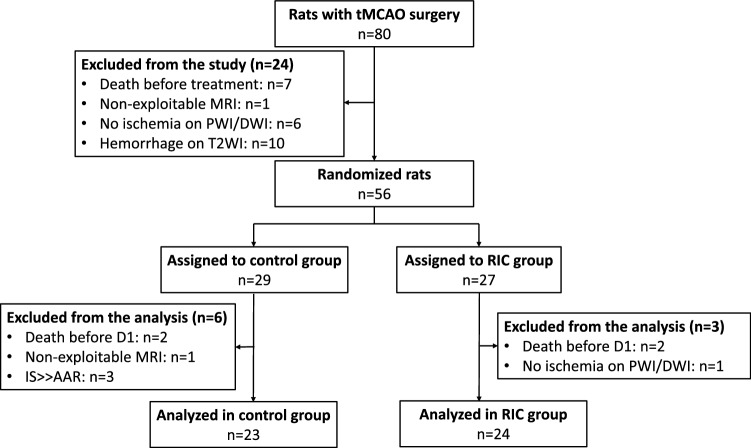
CONSORT-like diagram of the overall study. *CONSORT* Consolidated Standards of Reporting Trials, *AAR* area at risk, *D1* day 1, *DWI* diffusion-weighted imaging, *IS* infarct size, *MRI* magnetic resonance imaging, *PWI* perfusion-weighted MRI, *tMCAO* transient middle cerebral artery occlusion, *T2WI* T2-weighted imaging, *RIC* remote ischemic conditioning.

The study was performed from July 2018 to May 2019 at the University of Lyon, using the MRI facilities of CERMEP, and from February 2019 to June 2019 at the Hatter Cardiovascular Institute, using the MRI facilities of King’s College London. Figure 1 shows the CONSORT-like chart of the pooled data. In total, 47 animals were analyzed after applying all the exclusion criteria (n = 23 in the control group and n = 24 in the RIC group). Supplementary Fig. S3 and Fig. S4 show the CONSORT-like charts of the studies from each centre.

**Figure 2:**
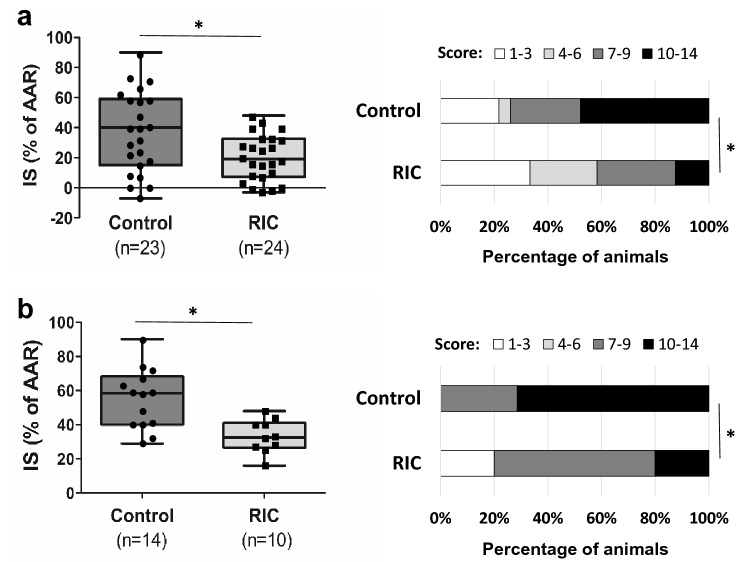
Efficacy outcome. Shown are the boxplots and individual data of primary endpoint [infarct size (IS) as a percent of area at risk (AAR) as determined with magnetic resonance imaging] and the distribution of neuroscores at 24 h (ranging from 0 to 14, with higher scores indicating more severe disability) in the control and remote ischemic conditioning (RIC) groups for all the included animals (panel (**a**)) and for subgroup of rats with corticostriatal apparent diffusion coefficient per-occlusion lesions (panel (**b**)). *p < 0.05.

Baseline data (ADC lesion and AAR) did not significantly differ between groups; however, there was a trend towards smaller ADC lesions in the RIC group before treatment (Table 1). IS as % of AAR was significantly reduced in the RIC group (Table 1; Fig. 2a). In univariate analysis, belonging to the RIC group explained an IS decrease of 17% of the AAR (− 17% [− 30%; − 4%], *p* = 0.01). Because the differences in ADC lesion size at baseline may explain in part the differences in final IS, we performed a multivariate analysis using ADC(%AAR) and treatment group as explanatory variables of IS(%AAR). As expected, ADC(%AAR) was a strong predictor of IS(%AAR) (67% [54%; 79%], *p* < 0.001). However and importantly, RIC group remained independently and significantly associated with the infarct-limiting effect (− 7% [− 14%; − 0.02%], *p* = 0.049). RIC had no statistically significant effect on %HSE or infarct growth, although there was a trend in reduction for each parameter (Table 1). In line with IS reduction, RIC significantly improved neuroscores (Table 1; Fig. 2a).

**Table 1:** Baseline characteristics and efficacy outcome for all the included animals. Data are presented as median [25th percentile; 75th percentile].

	Baseline MRI data			Primary endpoint	Secondary endpoints		
	MRA	ADC (%HH)	AAR (%HH)	IS (%AAR)	%HSE (%)	Infarct growth (mm^3^)	Neuroscores
Control (n = 23)	0 [0; 1]	25 [6; 32]	45 [36; 54]	40 [17; 59]	8 [5; 15]	26 [5; 93]	9 [7; 11]
RIC (n = 24)	0 [0; 1]	9 [4; 25]	48 [31; 53]	19* [8; 32]	5 [3; 9]	11 [− 8 ; 33]	6* [3; 8]
*p*	0.801	0.202	0.670	0.028	0.136	0.077	0.032

*AAR* area at risk, *ADC* apparent diffusion coefficient, *%HH* percentage of healthy hemisphere, *%HSE* hemispheric space-modifying effect of edema, *IS* infarct size, *MRA* magnetic resonance angiography, *MRI* magnetic resonance imaging, *RIC* remote ischemic conditioning.

*p < 0.05.

The measurements of the same MRI variables obtained by two researchers demonstrated an excellent agreement for ADC lesion, %HSE, uncorrected or edema-corrected T2WI lesion, and for the composite primary endpoint IS(%AAR), and a good agreement for AAR and infarct growth (Table 2).

**Table 2:** Agreement of imaging variables measurements between two independent investigators (Intra Class Coefficient or ICC).

	ADC (DWI)	AAR (PWI)	LVu (T2WI)	%HSE (T2WI)	LVc (T2WI)	IS (%AAR)	Infarct growth
mm^3^	88%	79%	99%	N/A	97%	N/A	80%
%HH (except IS)	89%	76%	99%	96%	96%	92%	N/A

*AAR* area at risk, *ADC* apparent diffusion coefficient, *DWI* diffusion-weighted imaging, *IS* infarct size, *LVc* lesion volume corrected, *LVu* lesion volume uncorrected, *N/A* non-applicable, *PWI* perfusion-weighted imaging, *T2WI* T2-weighted imaging.

There was an excellent agreement between hemispheric edema-corrected lesion volume (%HLVc), i.e. IS expressed as percentage of hemisphere, measured by T2WI and TTC staining (ICC = 87%). The analysis of brain slices after TTC staining revealed the reduction of IS in the RIC group (control: 17% [7–33%] vs RIC: 8% [6–20%]), in line with its MRI counterpart %HLVc (control: 19% [5–23%] vs RIC: 6% [2–16%]), although this difference did not reach significance (*p* = 0.204).

To evaluate the effect of treatment in the most severely injured animals (in analogy to ‘core-too-large’ patients^41^), we performed a subgroup analysis by including only the rats with ADC lesions > 100 mm^3^. This stratification led to groups with fewer individuals (n = 14 in the control group and n = 10 in the RIC group) but with ADC lesions and AAR that were balanced between the groups (Table 3). As for the overall population, the RIC effect was found significant again on both the primary endpoint and the neurofunctional secondary endpoint (Table 3; Fig. 2b).

**Table 3:** Baseline characteristics and efficacy outcome in the subgroup of rats with ADC lesion > 100 mm^3^. Data are presented as median (25th percentile; 75th percentile).

	Baseline MRI data			Primary endpoint	Secondary endpoints		
	MRA	ADC (%HH)	AAR (%HH)	IS (%AAR)	%HSE (%)	Infarct growth (mm3)	Neuroscores
Control (n = 14)	0 [0; 0]	32 [26; 34]	48 [39; 56]	58 [40; 66]	14 [11; 20]	58 [6; 136]	10 [9; 11]
RIC (n = 10)	0 [0; 0]	28 [18; 33]	54 [50; 55]	32* [27; 40]	9 [8; 16]	20 [− 23; 113]	8* [7; 9]
*p*	0.178	0.380	0.219	0.004	0.198	0.349	0.016

*AAR* area at risk, *ADC* apparent diffusion coefficient, *%HH* percentage of healthy hemisphere, *%HSE* hemispheric space-modifying effect of edema, *IS* infarct size, *MRA* magnetic resonance angiography, *MRI* magnetic resonance imaging, *RIC* remote ischemic conditioning.

*p < 0.05.

## Discussion

This study was performed in the wake of another preclinical, randomized, controlled, multicentre international trial^42^, studying anti-CD49d treatment for acute brain ischemia. We aimed to investigate RIC as a potential method to reduce irreversible brain damage in subjects with an acute ischemic stroke in adjunct to reperfusion therapy, using a rat model. We designed our study to adhere as much as possible to the key tenets of the guidelines on preclinical stroke research, specifically with regard to the use of randomization, blinding, multi-laboratory approach and using longitudinal MRI^22,23^. The experiments were performed using the specific animal management and experimental conditions of each centre without overt standardization as a mean to improve the reproducibility of study results^43^.

### Area at risk

In addition to the issue of compliance with experimental guidelines, another important factor which can compromise the investigation of infarct size (IS) is the variation in ischemic brain tissue volumes between animals, which necessitates sufficiently large experimental groups. This variation stems largely from the variability of the anatomy of collaterals in the brain. Occlusion of the ostium of the middle cerebral artery can provoke infarct in different volumes of brain tissue—from minimal striatum to more than half of a hemisphere. For this reason, the true infarct-limiting effect of treatment can only be evaluated if IS is normalized with a parameter that takes into account this disparity. In the present study, we have decided to express IS as % of the ischemic ‘area at risk’. This method of data analysis is routinely used in preclinical rodent models of acute myocardial infarction, where AAR is demarcated by perfusing the heart with a dye (typically Evan’s blue) after coronary reocclusion^44^. However, the accurate measurement of AAR is more technically challenging in animal models of ischemic stroke. Importantly, the AAR may be estimated by advanced imaging techniques, such as high-resolution MRI, performed during the artery occlusion. An important feature of our study, therefore, was the use of high-resolution MRI to estimate our primary endpoint, i.e. IS as % of AAR.

### Infarct growth

The endpoints that are usually evaluated in pre-clinical and clinical studies of ischemic stroke as imaging outcomes are IS, infarct growth or penumbral salvage^19,41,45^. Although the two latter do account for variability across patients through the measurement of ischemic core at admission, they are both based on the assumption that the ADC lesion measured at baseline is a good estimate of the ADC lesion present at the time of reperfusion. However, there is a non-negligible time-lapse between baseline DWI and reperfusion (~ 45 min in our pre-clinical set-up and ~ 90 min in the clinics)^41^ during which the ADC lesion is still growing^46^. In rat population as in patients, there are slow and fast progressors with regard to ADC growth rate^47^ (as reflected in our data by the variability of ADC lesion volumes, despite the fact that DWI was obtained roughly at the same time post-MCAO for all animals, i.e. ~ 45 min). Therefore, normalizing the final IS with per-occlusion ADC lesion volume amounts to normalizing with a moving target. Infarct growth during ischemia further depends on residual perfusion in the ischemic area, which differs among animals. This increases the standard deviations and probably explains in part why infarct growth was not statistically different between groups in our study. Such a result is not totally surprising, as sample size calculation was based on IS and not on infarct growth—in other words, this simply means that the use of infarct growth as an endpoint of efficacy outcome implies the need to increase sample size. Based on our result (means delta = 37, SD = 75), for a power of 0.8 and an alpha error of 0.05, the sample size would be 65 rats per group, hence a total of 216 operated rats to include 130 rats in the analysis. Therefore using the infarct growth endpoint would imply to increase the number of centres involved in the study. In contrast to ADC, the AAR is relatively stable in time, at least in our rat model^46^, while still reflecting the individual response to MCAO. When designing the study, the AAR therefore appeared to us as a more robust parameter than the ADC lesion for normalizing the final IS.

### Limitations

Our approach has several limitations. First, we performed a patient-level analysis (comparing lesion volumes at H0 and H24) whereas a voxel-level analysis may provide more accurate information about the fate of each tissue region differently affected by the ischemia during the occlusion (and in particular a more accurate estimation of penumbral salvage). For example, Hougaard et al.^17^ showed that RIC (per-conditioning) reduced the tissue risk of infarction after adjustment for baseline perfusion and diffusion lesion severity at the voxel level (while their patient-level analysis on infarct size did not show any effect on treatment). However, co-registration of H24 and H0 data sets is not a trivial task in our rat model because of the non-linear deformation induced by hemispheric edema (see midline shifts in Supplementary Fig. S1 and Fig. S2 online), and consequently the voxel-based approach may also be prone to errors. In future works, atlas-based registration may help addressing this issue^39^.

Secondly, the AAR was measured by manually delineating on the semi-quantitative perfusion maps. The accurate measurement of an arterial input function is challenging in rats because of partial volume effects, therefore the automatic approach used in patients to determine AAR (i.e. using a threshold on quantitative Tmax maps obtained after deconvolution of the brain tissue curve with an arterial input function) could not be applied here. Nevertheless, we obtained reproducible AAR compared to a previous study by the Lyon investigators using the same rat model of ischemic stroke^27^. In addition, inter-observer agreement for AAR was good, thus showing that manual contouring provided robust estimates. To go further, standardisation of the measurement of AAR by means of automatic post-processing procedures, and determination of thresholds obtained from permanent and transient MCAO, should be proposed in order to further improve the accuracy and reproducibility of the assessment of AAR.

Another limitation of the study is the duration of the reperfusion period. The extent of the in-vivo ischemic lesions in a rat model with 90-min ischemia is known to maximize at 48 h^48^. However, based on our previous experience^49^, extending the time of reperfusion beyond 24 h is accompanied by an increased mortality in the control group, as well as by an increased occurrence of visible hemorrhagic transformation of stroke in the survived animals. Either the death of animals, observed normally with the largest infarct volumes, or exclusion of animals because of the hemorrhages could end up with the smaller recorded IS than the real one is. As IS was the primary endpoint in our study, we chose to limit the reperfusion period to 24 h, which is standard for the experimental studies on ischemic stroke^20^.

In London the MCAO surgery, RIC, functional evaluation and TTC staining were all performed by the same researcher, which could be regarded as a limitation of the study. However, all the animals codes were concealed immediately after the procedure of RIC, ensuring that the researcher was blinded to treatment allocation. In addition, the exclusion rate was high, accounting for 30% in the London arm of the study and 45% in Lyon arm. Nevertheless, this only emphasises the importance of implementation of the exclusion criteria, based on high-resolution MRI, for preclinical studies on stroke.

### The importance of high-resolution MRI for pre-clinical studies in acute ischemic stroke

There are many benefits in performing per-occlusion MRI in pre-clinical neuroprotection studies: (1) the rigorous inclusion of animals; (2) the use of translational imaging endpoints to measure outcome; and (3) the comparison of baseline (pre-treatment) MRI data in order to make sure that ischemic areas were evenly distributed between groups. In the present study, AAR was similar between groups but, despite a rigorous randomization, there was a trend towards smaller ADC lesions ‘at admission’ in the RIC group. ADC lesion size is known to be a strong predictor of final lesion size, so in this case there was a concern that this imbalance in initial tissue damage may have influenced the final outcome in favour of the RIC treatment group. We therefore performed a multivariate analysis to evaluate if RIC treatment was still efficient all other things remaining equal. As expected, the final infarct size was strongly determined by the initial ADC lesion size. Importantly, the infarct-limiting effect of RIC was still observed in the multivariate analysis, thus showing that ADC imbalance was not the only explanation for smaller IS in the RIC group. Furthermore, the infarct-limiting effect of RIC was confirmed by the analysis of the subgroup of rats with corticostriatal lesions only (in which ADC lesion sizes were evenly distributed between the groups). Of note, this subgroup analysis, including rats with the largest ischemic cores, is of relevance for clinical translation, as it has been shown that ‘core-too-large’ patients, although may benefit from MT, particularly if they have substantial penumbral volumes^41^, are less likely to have a 3-month functional independence and therefore may benefit the most from neuroprotection therapy (only 39% of ‘core-too-large’ patients treated with thrombectomy are independent at 90 days post-stroke vs 72% of ‘core-not-too-large’ patients^41^)^50^.

The gold standard for measuring lesion sizes in pre-clinical studies of neuroprotection is TTC staining. As expected, there was a good agreement between IS measured by TTC and MRI. However, the difference between control and RIC groups did not reach significance with the TTC endpoint. Again, this highlights the need for increasing sample size (and hence probably the number of centres). Based on our results (mean delta of 7, SD of 14), for a power of 0.8 and an alpha error of 0.05, the sample size would need to be 63 rats per group, i.e. a total of 210 operated rats to include 126 animals in the analysis. Of note, this is assuming that all the included animals have had an ischemia, which may be more difficult to ascertain without per-occlusion MRI (the conventional use of Laser Doppler Flowmetry having its own limitations)^51^. The additional advantage of using an MRI measurement of IS is that the brain may then potentially be used for evaluating mechanistic hypothesis rather than lesion size only, thus further refining the experiments.

### Functional outcome

Regarding the behavioural neurological evaluation, we used a more advanced scale in comparison with previously described scales for evaluating the effect of RIC on functional outcome in an animal stroke model^20^. This was a 0–22 scoring scale, incorporating three previously reported scales^32–35^. Importantly, our two-centre study showed that infarct-limiting effects of RIC were associated with an improved functional outcome, both in the overall population and in the subgroup analysis.

### Potential impact

Altogether these results suggest that RIC treatment performed at reperfusion is safe and effective in improving both morphological and functional outcomes in a rat model of ischemic stroke. There was also a trend towards reducing hemispheric edema, although this may just be related to the fact that smaller infarcts are associated with smaller hemispheric edema.

Until recently, RIC was regarded as the intervention with the best evidence for cardioprotection in patients with acute myocardial infarction^52,53^. However, the positive effects of RIC in these patients appear to be partially or completely masked by the medication commonly administered in the acute phase^54,55^, such as beta-blockers^56^ and antiplatelet therapy^57,58^, especially the third-generation P2Y12 antagonists^59,60^. This might explain in part the neutral results of the latest multicentre clinical trial^61^. Conversely, in patients with acute stroke undergoing reperfusion therapy, beta-blockers are not routinely administered, and antiplatelet treatment, normally confined to aspirin or clopidogrel, is generally introduced secondarily, especially in those treated with alteplase^2^. For these reasons, the potential benefit of using RIC in patients with ischemic stroke may be higher than in the setting of myocardial infarction. In the two completed clinical trials^17,19^, patients were included provided they had acute ischemic stroke irrespectively of the presence of an artery occlusion or reperfusion therapy. Additionally, populations were heterogeneous in terms of baseline lesion size and AAR, as well as in terms of reperfusion therapy (MT was only used in about one third of patients in the RESCUE-BRAIN study)^19^. This justifies further preclinical and clinical studies, aimed at investigating the effects of RIC in the setting of an acute stroke, with improved patient selection and improved clinical endpoints selection. Our two-centre international study is an important step underpinning further research in this direction. The obtained results, showing IS limitation and improvement of functional outcome by RIC, are in agreement with the results of previously published experimentsl studies^20,21^. However, our study was the first one to employ clinically relevant endpoints. Specifically, we evaluated infarct growth over 24 h, as well as IS as % of the AAR. In addition, we aimed at the highest possible quality in the methodology of the study, by rigorously following the existing guidelines on preclinical stroke research^22,23^, including the recommendations on international collaboration^24,25^.

The studies on the mechanisms of RIC-induced *neuroprotection* are quite limited in comparison with the amount of studies on the mechanisms of RIC-induced cardioprotection (reviewed in^62^). Specifically, the role of RIC in the reduction of oxidative stress, inflammation, and supression of ischemia-induced neuronal apoptosis in the penumbra area has been reported (reviewed in^20^). Further investigation of the neuroprotective mechanism of RIC is warranted to establish the most effective and safe implementation of this phenomenon into clinical practice. As evidenced by the current study, the rigorous methodological approach, including the use of clinically relevant endpoints, is highly advisable in future studies on the mechanisms of RIC, as it can improve the robustness of results.

## Conclusion

RIC in the setting of acute ischemic stroke in rats is safe, reduces infarct size and improves functional recovery in a two-centre international study using translational imaging endpoints.

## Supplementary information

Supplementary Information.

## Data Availability

The full data set obtained from this study is publicly available at the figshare repository—https://figshare.com (https://doi.org/10.5522/04/10529867).
