# Supplementary material for: Neuroprotection by remote ischemic conditioning in the setting of acute ischemic stroke: a preclinical two-centre study
Source: Sci Rep. 2020 Oct 9;10:16874. doi: 10.1038/s41598-020-74046-4 (PMC7547701; doi:10.1038/s41598-020-74046-4)
Supplement: Supplementary file 1 — Supplementary Information. [file 41598_2020_74046_MOESM1_ESM.pdf]

SUPPLEMENTARY INFORMATION

Scientific Reports

Neuroprotection by remote ischemic conditioning in the setting of acute ischemic stroke: a preclinical two-centre study

Basalay MV[1¥], MD, PhD, Wiart M[2¥], PhD, Chauveau F[3], PhD, Dumot Ch[2], MD, MSc, Leon Ch[2], MSc, Amaz C[4], MSc, Bolbos R[5], PhD, Cash D[6], PhD, Kim E[6], PhD, Mechtouff L[7], MD, MSc, Cho TH[7], MD, PhD, Nighoghossian N[7], MD, PhD, Davidson SM[1], PhD, Ovize M[2ǂ], MD, PhD, Yellon DM[1*ǂ], PhD, DSc, FRCP.

Affiliations

[1] The Hatter Cardiovascular Institute, University College London, 67 Chenies Mews, London WC1E 6HX, United Kingdom

[2] Université Lyon, CarMeN Laboratory, INSERM, INRA, INSA Lyon, Université Claude Bernard Lyon 1, 69003, Lyon, France

[3] Université Lyon, Lyon Neuroscience Research Center, CNRS UMR5292, Inserm U1028, Université Claude Bernard Lyon 1, Lyon, France

[4] Clinical Investigation Center, CIC 1407, HCL, Louis Pradel Hospital, Lyon, France

[5] CERMEP-Imagerie du Vivant, Bron, France

[6] Department of Neuroimaging, Institute of Psychiatry, Psychology and Neuroscience, King's College London, James Black Centre, 125 Coldharbour Lane, London, SE5 9NU, United Kingdom

[7] Stroke department, Université Claude Bernard Lyon 1, CREATIS CNRS UMR 5220-INSERM U1206, INSA-Lyon; Hospices Civils de Lyon, Lyon, France

[¥] Co-first authors

[ǂ] Co-last authors

*Corresponding author: Prof Derek Yellon

d.yellon@ucl.ac.uk

Phone: +44 203 447 9888

The Hatter Cardiovascular Institute

University College London

67 Chenies Mews

London WC1E 6HX

United Kingdom

Supplementary Tables

	London	Lyon
**Field strength**	9.4T	7T
**Gradients**	660 mT/m	440 mT/m
**Hardware system**	Bruker BioSpec	Bruker BioSpec
**Software system**	ParaVision 6.0.1	ParaVision 5.1
**Volume coil for emission: inner diameter (mm)**	86	72
**Surface coil for reception: diameter (mm)**	32	25

Table S1 - MR system characteristics in each center

	**T2WI: 2D RARE sequence** (RARE factor 8)		**MRA: 2D-inflow angiography with flow compensation**		**DWI: 2D diffusion-weighted echo planar imaging (EPI)**		**PWI: 2D dynamic susceptibility contrast-enhanced (DSC)-MRI**	
**MR imaging parameters**	**London**	**Lyon**	**London**	**Lyon**	**London**	**Lyon**	**London**	**Lyon**
TE/TR (ms/ms)	75/3500	75/5000	3/18	4.1/18	26.26/5000	23.2/5000	11.1/600	8.2/600
b values (s/mm^2)	N/A	N/A	N/A	N/A	0/1500/3000	0/1500/3000	N/A	N/A
Flip angle (degrees)	180	180	90	90	90	90	25	50
Number of averages	2	2	2	3	2	2	1	1
Bandwidth (kHz)	37	35	100	50	300	300	300	247
Field of view (mm^2)	35 x 35	35 x 35	35 x 31	35 x 25	35 x 35	35 x 35	35 x 35	35 x 35
Slice thickness/interslice (mm)	1/0	1/0	0.4/0.15	0.4/0.25	1/0	1/0	1/0	1/0
Number of slices	15	15	60	60	15	15	15	15
Matrix size	256 x 256	256 x 256	256 x 226	256 x 184	128 x 128	128 x 128	80 x 80	80 x 80
Number of repetitions	N/A	N/A	N/A	N/A	N/A	N/A	100	100
Contrast agent CA (i.v., bolus)	N/A	N/A	N/A	N/A	N/A	N/A	Gd-DO3A-butrol	Gd-DOTA
Dose (ml/kg)	N/A	N/A	N/A	N/A	N/A	N/A	0.6	0.6
Acquisition time (min, sec)	3 min 44 sec	4 min 44 sec	8 min 8 sec	6 min 27 sec	4 min 40 sec	4 min 40 sec	1 min	1 min

Table S2 - MR imaging protocol characteristics in each center

TE: echo time; TR: repetition time; T2WI: T2-weighted MRI; MRA: magnetic resonance angiography; DWI: diffusion-weighted imaging; PWI: perfusion-weighted imaging; N/A: not applicable; Gd-DO3A-butrol: Gadovist (Bayer Healthcare, Germany); Gd-DOTA: DOTAREM (Guerbet, France).

Sign	Description	Score
**Motility,<br>spontanous activity**	Normal	0
	Slightly reduced exploratory behaviour	1
	Moving limbs without proceeding	2
	Moving only to stimuli	3
	Unresponsive to stimuli, normal muscle tone	4
	Premortal signs, severe hypotonia	5
**Gait**	Straight walking	0
	Walking toward controlateral side	1
	Alternate circling & straight walk	2
	Alternate circling & walking toward paretic side	3
	Circling/other gait disturbances	4
	Constant circling toward paretic side	5
**Postural signs**	Degree of forelimb flexion when held by tail	0-2
	Degree of body rotation when held by tail	0-2
**Parachute reflex**	Symmetrical	0
	Asymmetrical	1
	Controlateral forelimb retracted	2
**Lateral resistance**	Degree of resistance against lateral push	0-2
**Limb placing**	Ipsilateral forelimb: normal, weak, no placing	0-2
	Controlateral forelimb: normal, weak, no placing	0-2
**TOTAL**		**0-22**

Table S3 - Neuroscore rating

Supplementary Figures and Figure Legends

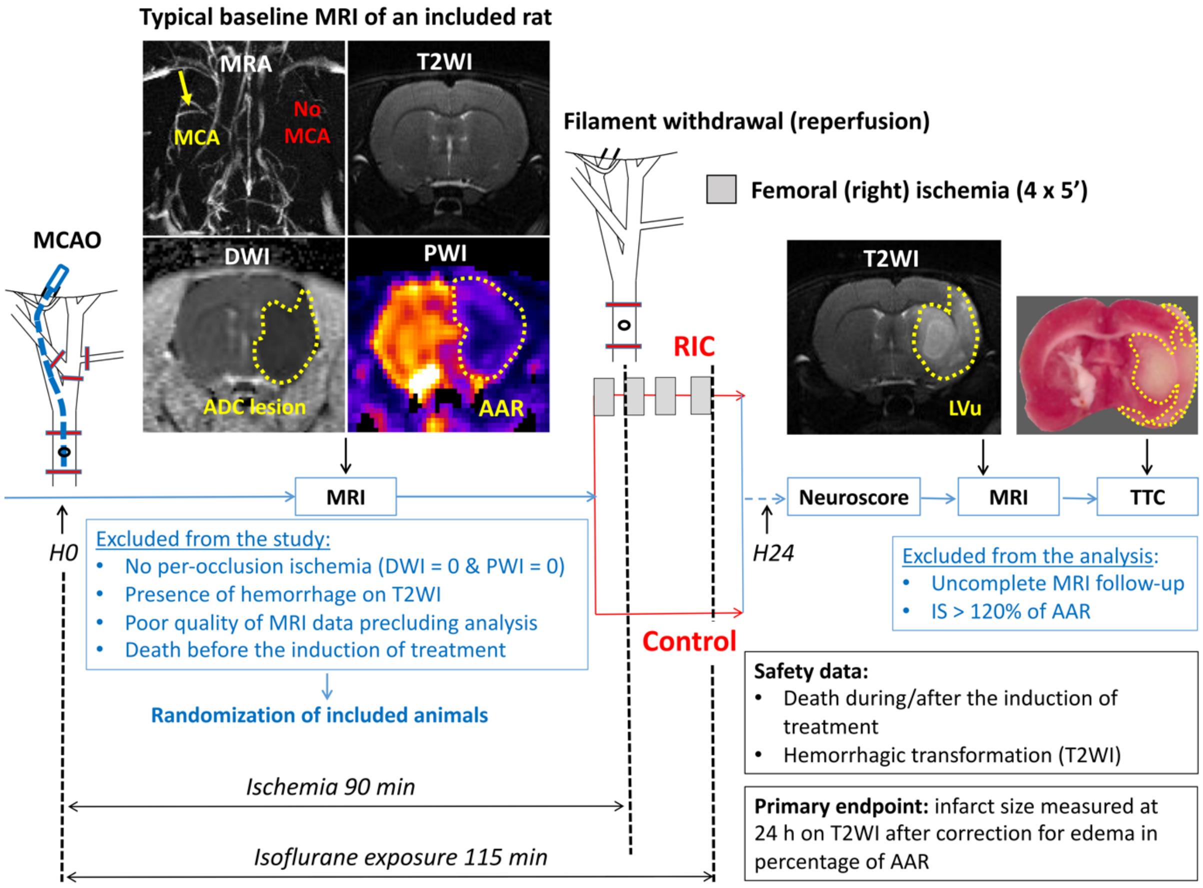

Figure S1 - Study design.

AAR: area at risk; ADC: apparent diffusion coefficient; DWI: diffusion-weighted imaging; H0: baseline time point; H24: 24 hours; IS: infarct size; LVu: lesion volume uncorrected; MCA: middle cerebral artery; MCAO: middle cerebral artery occlusion; MRA: magnetic resonance angiography; MRI: magnetic resonance imaging; PWI: perfusion-weighted imaging; RIC: remote ischemic conditioning; TTC: triphenyl tetrazolium chloride; T2WI: T2-weighted imaging.

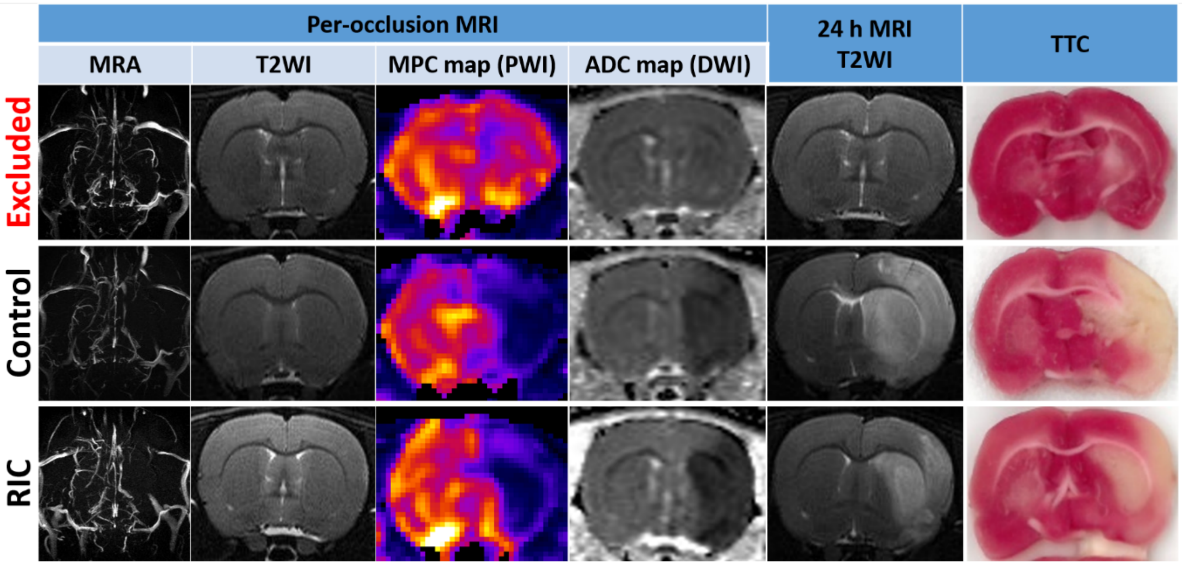

Figure S2 - MRI and TTC data of representative rats.

Only one slice is shown from the 3D data sets. First row: one animal illustrating the inclusion/exclusion criteria: here both PWI and DWI are negative, meaning there is no cerebral ischemia. The MRA score was 2 (indicative of ipsilateral middle cerebral artery patency). This rat was wrongly included at admission and then excluded from the analysis (as shown on the CONSORT-like diagram). As expected, no infarct was present on follow-up T2WI and TTC staining. Second and third rows: animals from the control and RIC groups respectively. These two rats presented with the same per-occlusion MRI features: MRA score of 0 (indicative of total MCA occlusion), AAR of 40% (control) and 50% (RIC) of the hemisphere, corticostriatal ADC lesion of 34% (both control and RIC). At 24h, the infarct has grown up at the expense of the penumbra in the control animal (50%), while it remained within the ADC lesion borders in the animal treated with RIC (29%).

ADC: apparent diffusion coefficient; DWI: diffusion-weighted imaging; MPC: maximum peak concentration; MRA: magnetic resonance angiography; MRI: magnetic resonance imaging; PWI: perfusion-weighted MRI; RIC: remote ischemic conditioning; TTC: triphenyl tetrazolium chloride; T2WI: T2-weighted imaging.

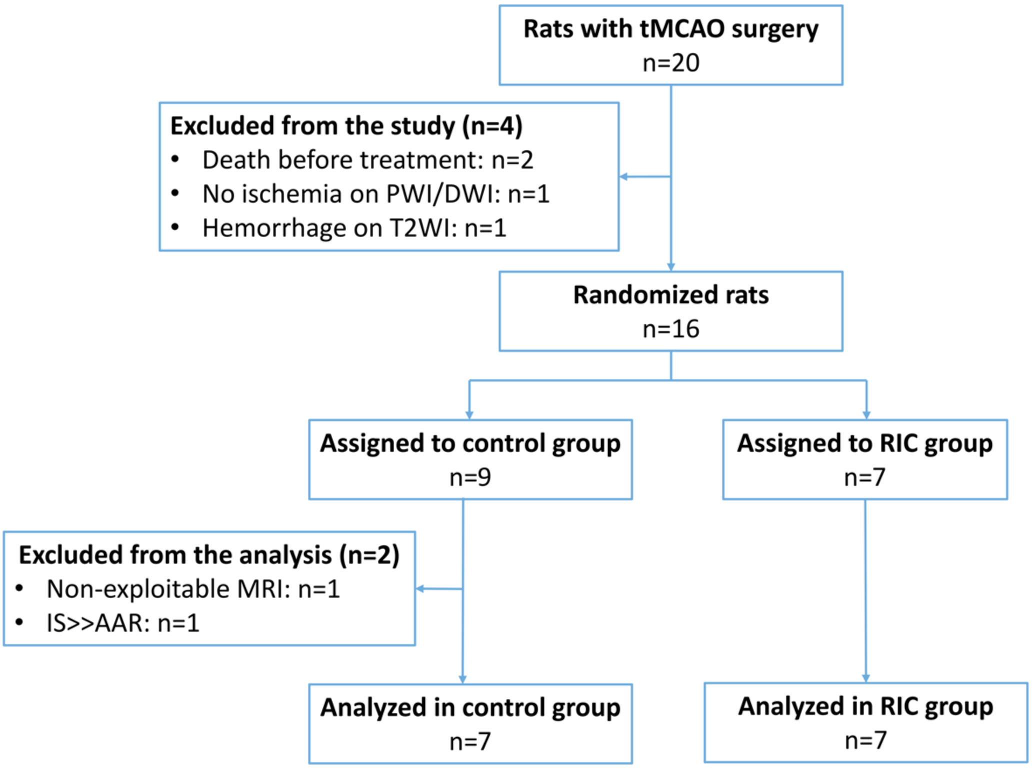

Figure S3 - CONSORT-like diagram of London study.

CONSORT = Consolidated Standards of Reporting Trials; tMCAO: transient middle cerebral artery occlusion; PWI: perfusion-weighted imaging; DWI: diffusion-weighted imaging; T2WI: T2-weighted imaging; RIC: remote ischemic conditioning; MRI: magnetic resonance imaging; IS: infarct size; AAR: area at risk.

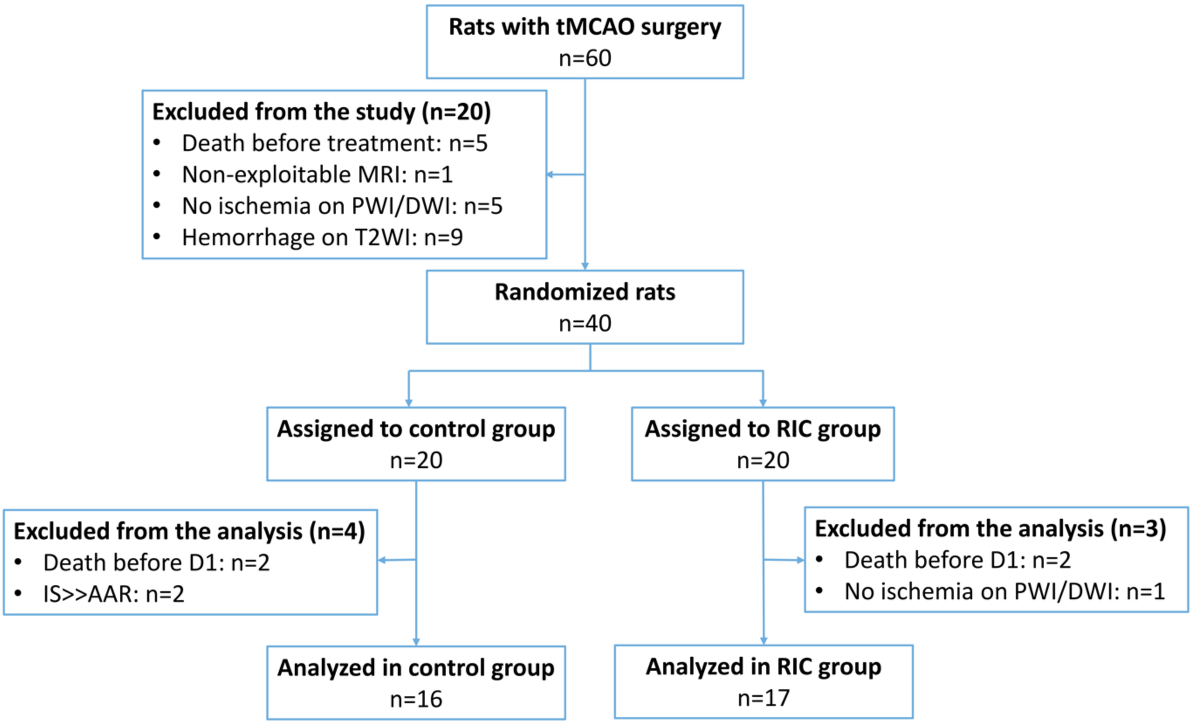

Figure S4 - CONSORT-like diagram of Lyon study.

CONSORT = Consolidated Standards of Reporting Trials; tMCAO: transient middle cerebral artery occlusion; MRI: magnetic resonance imaging; PWI: perfusion-weighted imaging; DWI: diffusion-weighted imaging; T2WI: T2-weighted imaging; RIC: remote ischemic conditioning; IS: infarct size; AAR: area at risk; D1: day 1 post-tMCAO.
